# On the Mental Health Needs Under the Russian Invasion in Ternopil, Ukraine: A Preliminary Report on the Provision of Mental Health Service

**DOI:** 10.5334/aogh.4076

**Published:** 2023-06-08

**Authors:** Mizuki Hino, Yasuto Kunii, Bogdan I. Gerashchenko, Yumiko Hamaie, Shinichi Egawa, Shinichi Kuriyama, Oksana O. Shevchuk, Mykhaylo M. Korda, Olena P. Venher, Hiroaki Tomita

**Affiliations:** 1 Department of Disaster Psychiatry, International Research Institute of Disaster Science, Tohoku University, Miyagi, Japan; 2 R.E. Kavetsky Insitute of Experimental Pathology, Oncology and Radiobiology, National Academy of Sciences of Ukraine, Kyiv, Ukraine; 3 International Cooperation for Disaster Medicine Lab., International Research Institute of Disaster Science, Tohoku University, Miyagi, Japan; 4 Department of Disaster Public Health, International Research Institute of Disaster Science, Tohoku University, Miyagi, Japan; 5 Department of Pharmacology and Clinical Pharmacology, I. Horbachevsky Ternopil National Medical University, Ternopil, Ukraine; 6 Department of Medical Biochemistry, I. Horbachevsky Ternopil National Medical University, Ternopil, Ukraine; 7 Department of Psychiatry, Narcology and Medical Psychology, I. Horbachevsky Ternopil National Medical University, Ternopil, Ukraine; 8 Department of Psychiatry, Graduate School of Medicine, Tohoku University, Miyagi, Japan

**Keywords:** mental health, CBRNE disaster, volunteer activities, resilience and sustainability

Russian armed forces launched a large-scale invasion of Ukraine on February 24, 2022. Civilian casualties continue to multiply. Over 15 million civilians have been displaced across Ukraine or abroad. Thus, the conflict threatens to become the worst humanitarian crisis since World War II. Military attacks on houses and civilian installations are still ongoing in Ukraine. Transport routes have been disrupted, threatening the lives and livelihoods of many civilians. The humanitarian and psychological crises in areas close to and far from the combat zone had worsened during the winter of 2023, with repeated bombings of civilian infrastructure. Russian attacks had led to power and water shortages in many parts of Ukraine, resulting in frequent blackouts and posing a significant risk to nuclear power plant operations. Moreover, there are concerns about the use of chemical weapons and the possibility of intentional or accidental nuclear and radiological damage.

Thus, the current situation in Ukraine faces a serious, complex, and continuing CBRNE (chemical, biological, radiological, nuclear, and high-yield explosives) disaster [1]. The mental health consequences of CBRNE disaster are more complex and severe than those experienced in natural disasters. Comprehensive assessment beforehand is even more crucial for mental health care to individuals affected by CBRNE disasters; however, under the ongoing CBRNE disasters, assessment methods are often limited. Although information on the present situation in Ukraine is limited, there are concerns about the worsening of mental health status [2,3]. Hence, this report was formulated from a communication between former colleagues in Ukraine and Japan, on the assessment of the actual situation of the mental health services provision in Ukraine under the threat of CBRNE hazard posed by the 2022 Russian invasion.

The assessment items to summarize current mental health needs in Ukraine were created (Table 1), referring to the “Mental Health and Psychosocial Support in Emergency Setting (MHPSS)” [4] of the Inter-Agency Standing Committee (IASC). Also, items to summarize mainly current mental health service resources including some changes before and after the invasion were created (Table 2), referring to the World Health Organization Assessment Instrument for Mental Health Systems (WHO-AIMS) version.2.2 [5]. The mental health workers (co-authors) at Ternopil National Medical University (TNMU) accumulated information relevant to the assessment items. The ethics committee of the International Research Institute of Disaster Science, Tohoku University, approved this report.

**Table 1:** The assessment item referring to the Mental Health and Psychosocial Support in Emergency Setting (MHPSS) in Inter-agency Standing Committee (IASC) and their answers by mental health workers at Ternopil National Medical University (TNMU).


The assessment item referring to the IASC-MHPSS and answers		

Type of information	Including	Answer

Experience of the emergency	People’s experiences of the emergency (perceptions of events and their importance, perceived causes, expected consequences)	Due to the war, emergency psychiatric care has changed, as most hospitals have been partially restructured and loaded with wounded. Assistance in acute mental conditions is provided immediately inpatient, outpatient or using the possibilities of telemedicine.

Mental health and psychosocial problem	Signs of psychological and social distress, including behavioural and emotional problems (e.g., aggression, social withdrawal, sleep problems) and local indicators of distress	post-traumatic stress disorder, anxiety, panic reaction, psychogenic agitation, sleep disorder, stupor, acute psychotic condition

	Signs of impaired daily functioning	sleep problems, inability to perform routine activities

	Disruption of social solidarity and support mechanisms (e.g., disruption of social support patterns, familial conflicts, violence, undermining of shared values)	internal migration, loss of places of living, loss of job, death of family members and friends, difficulties in educational area

	Information on people with severe mental disorders (e.g., through health services information systems)	N/A

Existing sources of psychosocial well-being and mental health	Ways people help themselves and others, i.e., ways of coping/healing (e.g., religious or political beliefs, seeking support from family/friends)	volunteer psychological centers, community crisis centers

	Ways in which the population may previously have dealt with adversity	personal psychotherapists, self psychological education, social and family support

	Types of social support (identifying skilled and trusted helpers in a community) and sources of community solidarity (e.g., continuation of normal community activities, inclusive decision-making, inter-generational dialogue/respect, support for marginalised or at-risk groups)	N/A


**Table 2:** The assessment item referring to the World Health Organization Assessment Instrument for Mental Health Systems (WHO-AIMS) and their answers by mental health workers at TNMU.


Item 2.10.3	Availability of medicines in mental health outpatient facilities	Answer

DEFINITION	Proportion of *mental health outpatient facilities* in which at least one psychotropic medicine of each therapeutic category (antipsychotic, antidepressant, mood stabilizer, anxiolytic and antiepileptic medicines) is available in the facility or in a nearby pharmacy all year long.	

MEASURE	Proportion: UN = unknown; NA = not applicable	0/1

NUMERATOR	Number *of mental health outpatient facilities* in which at least one psychotropic medicine of each therapeutic category is available in the facility or in a nearby pharmacy	

DENOMINATOR	Total number of *mental health outpatient facilities* (#)	

**Item 4.1.4**	**Staff working in or for mental health outpatient facilities**	**Answer**

DEFINITION	Number of full-time or part-time mental health professionals working in or for *mental health outpatient facilities*	

MEASURE	Number of mental health professionals: 1. *Psychiatrists* 2. Other *medical doctors*. not specialized in psychiatry. 3. *Nurses* 4. *Psychologists, social workers*, and *occupational therapists* 5. *Other health or mental health workers* Number; UN = unknown	12 (Ternopil) 0 9 3 0

NOTES	Include mental health staff working in government-administered outpatient facilities, NGO outpatient facilities and for-profit mental health outpatient facilities. Exclude professionals engaged exclusively in private practice.	

*83 (for the whole Ternopil region, including Ternopil). only 70 are occupied as of today		

**Item 4.1.5**	**Staff working in community-based psychiatric inpatient units**	**Answer**

DEFINITION	Number of full-time or part-time mental health professionals working in *community-based psychiatric inpatient units* per *bed*	

MEASURE	Number of mental health professionals: 1. *Psychiatrists* 2. Other *medical doctors*. not specialized in psychiatry. 3. *Nurses* 4. *Psychologists*, *social Markers*, and *occupational therapists* 5. *Other health or mental health Markers* Number of mental health professionals per *bed*; UN = unknown; NA = not applicable	0.04 0.04 0.4 0.006

NUMERATOR	Number of mental health professionals	61

DENOMINATOR	Number of *beds* in *community-based psychiatric inpatient units* (#)	630

**Item 2.9.1**	**Availability of psychosocial interventions in mental hospitals**	**Answer**

DEFINITION	Percentage *of patients* who received one or more *psychosocial interventions* in mental hospitals in the Iasi year	D = the majority (51 – 80%)

MEASURE	A = none (0%) B = a few (1 – 20%) C = some (21 – 50%) D = the majority (51 – 80%) E = all or almost all (81 – 100%) UN = unknown; NA = not applicable	

**Item 2.9.1**	**Availability of psychosocial interventions in mental hospitals**	**Answer**

NOTES	*Psychosocial intervention* sessions should last a minimum of 20 minutes to be counted for this item. Examples of psychosocial treatments include psychotherapy, provision of social support, counselling, rehabilitation activities, interpersonal and social skills training, and psychoeducational treatments. Do not include intake interviews, assessment, and follow-up psychopharmacology appointments as psychosocial interventions.	

**Item 2.9.3**	**Availability of psychosocial interventions in mental health outpatient facilities**	**Answer**

DEFINITION	Percentage of *users* who received one or more *psychosocial intervention* in *mental health outpatient facilities* in the last year	D – the majority (51–80%)

MEASURE	A = none (0%) B = a few (1 – 20%) C = some(21 – 50%) D = the majority (51 – 80%) E = all or almost all (81 – 100%) UN = unknown; NA = not applicable	

NOTES	*Psychosocial intervention* sessions should last a minimum of 20 minutes to be counted for this item. Examples of psychosocial treatments include psychotherapy, provision of social support, counselling, rehabilitation activities, interpersonal and social skills training, and psychoeducational treatments. Do not include intake interviews, assessment, and follow-up psychopharmacology appointments as psychosocial interventions.	

**Item 2.10.1**	**Availability of medicines in mental hospitals**	**Answer**

DEFINITION	Proportion *of mental hospitals* in which at least one psychotropic medicine of each therapeutic category (antipsychotic, antidepressant, mood stabilizer, anxiolytic and antiepileptic medicines) is available in the facility all year long	

MEASURE	Proportion; UN = unknown; NA = not applicable	0/1

NUMERATOR	Number *of mental hospitals* in which at least one psychotropic medicine of each therapeutic category is available	

DENOMINATOR	Total number *of mental hospitals* (#)	

NOTES	Include mental health staff working in government-administered *community-based psychiatric inpatient units*, NGO *community- based psychiatric inpatient units* and for-profit *communitybased psychiatric inpatient units*. Exclude professionals engaged exclusively in private practice.	

**Item 4.1.6**	**Staff working in mental hospitals**	**Answer**

DEFINITION	Number of full-time or part-time mental health professionals per *mental hospital bed*	

MEASURE	Number of mental health professionals:	
	1. *Psychiatrists* 2. Other *medical doctors*, not specialized in psychiatry. 3. *Nurses* 4. *Psychologists, social workers*, and *occupational therapists* 5. *Other health or mental health workers* Proportion: UN = unknown: NA = not applicable	29 per 630 beds 31 per 630 beds 257 per 630 beds 4 per 630 beds

NUMERATOR	Number of mental health professionals	

DENOMINATOR	Number of *mental hospital beds* (#)	

NOTES	Include mental health staff working in government-administered mental hospitals, NGO mental hospitals and for-profit mental hospitals. Exclude professionals engaged exclusively in private practice.	

**Item 6.2.2**	**Proportion of health research that is on mental health**	**Answer**

DEFINITION	Proportion of indexed publications that are on mental health in the last five years	

MEASURE	Proportion; UN = unknown	4/189

NUMERATOR	Total number of mental health publications on the country or region in the last five years as identified on PubMed	4

DENOMINATOR	Total number of health publications on the country or region in the last five years as identified on PubMed	6

NOTES	Studies need to involve respondents of the country or region. Investigators may be national or foreign researchers. The website of PubMed is: http://www.ncbi.nlm.nih.gov/entrez/query.fcgi	


Ternopil (estimated population: 225,004 in 2022) is a city located in the center of the Ternopil region (*oblast*) in western Ukraine, hosting many internally displaced persons (IDPs) from the eastern, northern, and southern combat zones of the country. Accordingly, the Volunteer Centre was established at TNMU, with students and staff involved in volunteer activities (https://vc.tdmu.edu.ua/). They also have been sending medicines and medical supplies to civilian and military hospitals, the Armed Forces of Ukraine, and territorial defense forces, to help IDPs in Ternopil (https://www.staffnet.manchester.ac.uk/news/display/?id=28436). Clinicians at TNMU have been volunteering at the free online health service, “Digital Clinic for Ukraine.” In addition, psychiatrists at TNMU provided mental health support to civilians, IDPs, and soldiers, by giving up holidays to treat them at local hospitals or offering psychological support to them via phone calls (https://www.tdmu.edu.ua/2022/02/28/fahivtsi-tnmu-nadayut-psyhologichnu-pidtrymku-naselennyu/).

In Tables 1 and 2, this report presents the details of TNMU’s responses to our assessment items. These responses, providing us with a vivid picture of the region’s mental health care system in this challenging situation, can be summarized as follows. As expected, mental health problems, such as post-traumatic stress reaction, anxiety, panic reaction, psychogenic agitation, sleep disorder, stupor, and acute psychotic conditions, were growing in this region. Although the Ternopil region is located far away from the front line, psychiatric beds had been reduced from 770 beds to 630 due to the functional reorganization of medical facilities to accommodate injured and sick patients. Consequently, the number of psychiatrists in the Ternopil region decreased from 83 before the invasion to 70. Meanwhile, several voluntary psychological centers were established around the TNMU, and the psychiatry department continues to provide inpatient and outpatient care, launching telemedicine services. Thus, the sustainability of local psychiatric care was maintained through compensatory activities such as volunteer center organizations and telemedicine.

While it is difficult to grasp the situation in a country under armed invasions or war, such as Ukraine in 2022, from the outside, it may be worthwhile to share information on the current mental health care needs and resources for the provision of care internationally. It may attract positive interest in the current situation and promote consideration of possible countermeasures. We believe that the use of globally recognized and unified tools such as IASC-MHPSS and WHO-AIMS in other regions of Ukraine as well as in Ternopil will be useful in acquiring precise information about the mental health needs for the entire country and developing future support under international coordination.
